# Noninterventional Study of Transdermal Fentanyl (Fentavera) Matrix Patches in Chronic Pain Patients: Analgesic and Quality of Life Effects

**DOI:** 10.1155/2015/198343

**Published:** 2015-03-11

**Authors:** Manuel Heim

**Affiliations:** ^1^Faculty of Biology, University of Freiburg, Schänzlestraße 1, 79104 Freiburg, Germany; ^2^MSL Consulting, Lujo Brentanos Strasse 11a, 83209 Prien, Germany

## Abstract

Fentanyl is considered to be an effective, transdermal treatment of chronic, cancer, and noncancer pain. This noninterventional, clinical practice-based study, on 426 patients attending 42 practices, assessed a proprietary, *Aloe vera*-containing, transdermal fentanyl matrix patch (Fentavera), for its analgesic effects, patients' quality of life (QoL) effects, tolerability, and adhesiveness. Study outcomes were mean changes from baseline of patient (11-point scales) and physician (5-point scales) ratings. After 1 and 2 months treatment, there were significant (*P* < 0.0001) decreases in patients' ratings of pain intensity, and impairment of walking, general activity, sleep quality, and QoL. For each parameter, the patient response rate was >30% at 2 months (response = 2-point decrease on 11-point rating scale). In a large majority of patients, the physicians rated the matrix patch as good or very good for analgesic effect, systemic and local tolerance, and adhesiveness. There were 30 adverse events in 4.2% of patients and analgesic comedications were reduced during treatment compared to before treatment. It is concluded, from this population-based data, that the proprietary, transdermal fentanyl matrix patch is effective and safe for chronic pain management in clinical practice, with significant positive analgesic and QoL effects, while being well tolerated and exhibiting good or very good adhesiveness.

## 1. Introduction

Chronic pain is defined as pain occurring for longer than several (3–6) months. Its incidence has been estimated as being close to 20% of the population and presents a large financial burden in terms of cost of treatment and loss of productivity and seriously affects the quality of lives of affected individuals [1–3]. The patient with chronic pain requires a combined program of self-treatment and primary and secondary (pain specialist) care, including both pharmacological and nonpharmacological treatment [1].

Opioids are already the mainstay of cancer pain treatment [4], with about 70% of cancer patients eventually requiring opioid treatment [5, 6]. In the treatment of moderate-to-severe noncancer pain, strong opioids are increasingly used [2, 7], with proven benefits in a variety of pain syndromes [6], including postoperative pain [8]. Opioids have become the most frequently used drugs for the treatment of chronic pain [1] and are a major component of the WHO-prescribed approach of stepwise escalation of analgesic treatment with increased pain intensity [9, 10]. Although the WHO-prescribed approach has been adopted, as intended, for treatment of chronic cancer pain, it has been, despite expert recommendations, only slowly adopted in the treatment of chronic noncancer pain, which mostly occurs in primary care [11, 12].

Fentanyl is a potent opioid and a treatment option at the top (step 3) of the WHO pain treatment ladder [13]. It is a high affinity agonist of the mu-opioid receptor, with 75–100-times greater analgesic potency than morphine [14]. Fentanyl has much greater lipid solubility than morphine [15], which means it has a greater accessibility to central nervous tissue. It has also been shown to have a skin permeability of several orders of magnitude greater than morphine [15], which makes fentanyl especially amenable to transdermal application.

Transdermal application of opioids has been developed to allow a long-term, continual, and stable level of analgesia, while avoiding the gastrointestinal adverse effects of oral opioids [16]. In prolonged opioid treatment, maintaining analgesia with minimal adverse effects requires minimal variation in opioid plasma levels. In this regard, slow transdermal opioid delivery from transdermal patches allows more effective analgesia and fewer adverse events than with oral or parenteral opioids, with lower rates of constipation, nausea, and sedation [17]. A meta-analysis of data derived from 8 prospective clinical studies, published between 1996 and 2004, and including 1,220 patients with cancer (*n* = 657) or noncancer pain (*n* = 563) showed that both fentanyl and morphine are effective by transdermal application [18], with improved pain scores after 28 days of treatment. The analgesic effect of transdermal fentanyl was also shown to be significantly greater than that of transdermal morphine. Other studies have demonstrated the effectiveness of transdermal fentanyl in children [19] and in the elderly [20]. Transdermal fentanyl provides a constant and sustained level of analgesia over several months [21] and may be used for more extended periods [22].

The adhesive polymer-matrix system of the transdermal patch used in this study differs from the standard reservoir transdermal patches and may be associated with a reduced risk of drug leakage. Matrix patches have been shown to be safe and efficacious in treating cancer pain [16, 23–25] and are often used to treat chronic noncancer pain [24, 25]. The aim of this multicentre noninterventional study was to perform a detailed assessment of transdermal fentanyl matrix patches in everyday clinical use. The main study outcomes were subjective assessments by the patients with additional subjective assessments made by the physicians. Patient-reported outcomes are now considered important in assessing treatment outcomes [26]. In addition to assessing analgesia, the aim was to assess other health-related factors such as mobility, sleep, and quality of life (QoL), which are now considered key outcomes in assessing pain management [27, 28]. The proprietary transdermal fentanyl (Fentavera) matrix patch was used throughout the study. The Fentavera matrix patch also contains an extract of* Aloe vera*, which is a herb that is widely-used in skin cosmetic products [29] and has been shown to have softening, hydrating effects on the skin [30]. The adhesiveness of the patch was also assessed.

## 2. Materials and Methods

### 2.1. Study Design

This was a multicentre, noninterventional, open-label study conducted in Germany. The objective of the study was to assess the effectiveness, in normal clinical practice, of a proprietary transdermal matrix patch containing fentanyl and an* Aloe vera* extract. The study aimed to include large number of adult patients with chronic pain that was responsive to opioid treatment. The effectiveness of transdermal fentanyl (Fentavera) matrix patches was assessed in terms of effects on the patients' pain intensity, their mobility, their sleep, and their QoL, using standard 11-point numerical rating scales. Additionally, the tolerability, safety, and adhesiveness of the patches were assessed using standardised questionnaires. The cause and location of the pain were recorded but were not inclusion or exclusion criteria. A fixed period of about six months (April 20, 2008, until October 31, 2008) was selected for recruitment. During this period, a total of 426 patients from 42 centres (from across the whole of Germany) were observed and included in the study.

### 2.2. Ethical Considerations

The study was conducted according to German law on medicinal products (AMG) [31], which states that a noninterventional clinical study is defined as a study of a licensed drug that is performed exclusively in clinical practices, without a clinical study protocol and using epidemiological methods (see AMG, article 4, paragraph 23) [31]. As such, the study is not a clinical study, as defined in AMG, article 4, paragraph 23 [31] and so requires no ethical approval. However, according to AMG, article 67, paragraph 6 [31], the study is registered at the German federal commission of physicians (KBV) and the head association of health insurances (GKV).

### 2.3. The Transdermal Matrix Patch

The transdermal patch that was used throughout the study was the product Fentavera (Acino Pharma AG, Switzerland), which is a matrix patch, not a reservoir patch, and is available containing a range of amounts of fentanyl base. The patch allows a slow, constant release and transdermal absorption of fentanyl, at a rate of 12, 25, 50, 75, or 100 microgram/hour [24] that is sustained for 3 days, after which the patch is replaced.

### 2.4. Patient Treatment

The fentanyl dosages received by the patients in this study varied, as would be expected according to the product label [32], which states that the required dose is dependent on the intensity of the pain and the patient's previous dosage of oral opioid treatment. According to the product label [32], the initial dosage of transdermal fentanyl is to be calculated from the patient's opioid dosage over the last 24 hours preceding the planned switch to transdermal fentanyl. This calculation uses the analgesic equivalence value of the opioid that was used immediately prior to switching to the matrix patch. The analgesic equivalence value is set relative to an oral dose of 30–40 mg morphine (listed in the product label information [32]) and is applied to convert the prior 24 hour dosage of opioid to the required initial dosage of transdermal fentanyl (Table 1).

### 2.5. Study Parameters

The study parameters, which were recorded at the start and after one and 2 months of treatment, were (i) pain intensity (type and origin of pain were recorded at the start of the study); (ii) fentanyl dosage; (iii) impaired mobility; (iv) impaired sleep; (v) impaired QoL; (vi) overall effectiveness of treatment; (vii) tolerance to the treatment, systemic and local (skin); (viii) adhesiveness of the matrix patch; (ix) comedications; (xi) adverse events (safety). The degree of algesia (pain intensity), impaired mobility (walking ability and general activity), impaired sleep, and impaired QoL (“lust for life” and mood) were subjectively rated by the patients, using an 11-point scale from no pain or impairment (=0) to most extreme pain or impairment (=10). The overall effectiveness, systemic and local (skin) tolerability, and adhesiveness of the patch were subjectively rated by the physician, using 5-point scales (very good, good, satisfactory, poor, and very poor).

The attending physician was requested to record all adverse events and was provided with forms for noting all the relevant details.

### 2.6. Statistical Method

The mean change from baseline of the patients' subjective point scores for each outcome was tested for statistical significance using comparison of the 95% confidence intervals (CI) of the mean change from baseline at one and 2 months.

## 3. Results

### 3.1. Patients

The patient demographics and their relevant clinical characteristics are listed in Table 2. A similar proportion of men and women were included and were of a broad range of adult ages. All patients had chronic pain, mostly derived from cancer or cancer treatment. Bone and nerve tissue were the most common sources of pain, with several patients having sources of pain in more than one tissue. All patients were selected on the basis of their pain being responsive to opioid treatment, including several (*n* = 119; 28%) patients who were opioid-naïve at the start of the study (see Table 1) and responded to the transdermal fentanyl matrix patch.

### 3.2. Treatment Dosages of Fentanyl

The fentanyl dose regimens varied between patients and for each patient as treatment progressed (see Table 3).

### 3.3. Analgesic Responses

**Figure 1:**
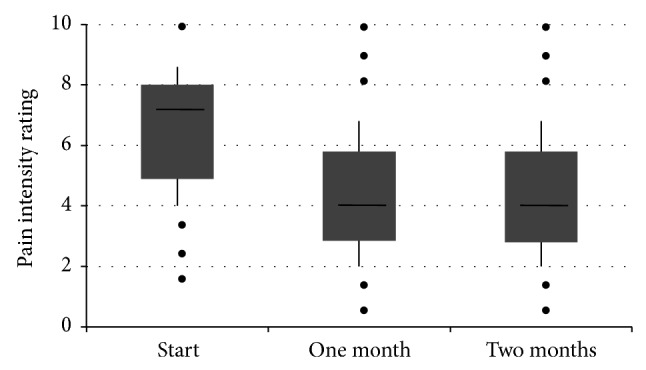
A box plot of the patients' rating of pain intensity at start of treatment and after one month and 2 months treatment. The data shows the median (horizontal bar), 1st and 3rd quartiles (top and bottom of box, resp.), and the standard deviation (vertical bar). The dots represent single outlying values.

After one and 2 months of treatment with transdermal fentanyl, the patients' subjectively assessed pain intensity was less than at baseline (Figure 1). At baseline, the mean level of pain intensity for the whole group of patients was 6.68 points. After one and 2 months treatment, the mean levels of perceived pain intensity were 4.39 and 3.59, respectively, and the mean changes from baseline were both highly significant (*P* < 0.0001; Table 4). The mean relative change in pain intensity was 33.4% and 44.9% at one month and 2 months, respectively. Using a change in the numerical rating scale of 2 units as being clinically significant [33], patient response rates of 66% and 72% were calculated at one month and 2 months, respectively. In the calculation of these values, patients with missing values were included in the denominator. Removal of these patients from the calculation provided values of 67% and 75%, respectively.

According to the subjective assessment of the physician, 68% had a “very good” or “good” analgesic response, while <4% had an “unsatisfactory” or “highly unsatisfactory” response.

### 3.4. Functional Responses

**Figure 2:**
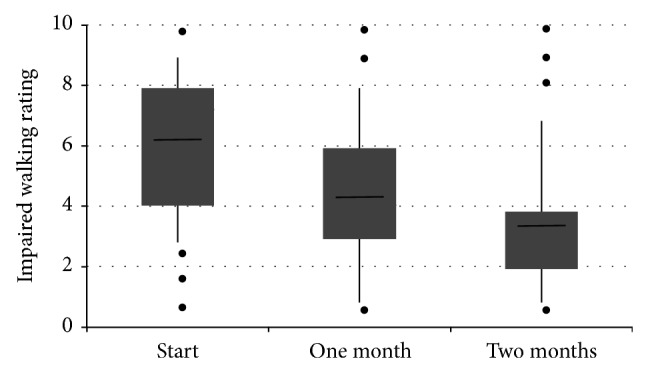
A box plot of the patients' rating of the impairment of walking at start of treatment and after one month and 2 months treatment. The data shows the median (horizontal bar), 1st and 3rd quartiles (top and bottom of box, resp.), and the standard deviation (vertical bar). The dots represent single outlying values.

Parallel to a significant reduction of pain intensity there were significant decreases in the patients' ratings of their impaired mobility. There were significant (*P* < 0.0001) decreases in the patients' rating of both their impaired walking ability (Figure 2, Table 4) and impaired general activity (Table 4), both after one month and after 2 months of treatment. The mean relative decreases in impaired functional parameters were above 30% at 2 months (Table 4).

### 3.5. Sleep and QoL Effects

**Figure 3:**
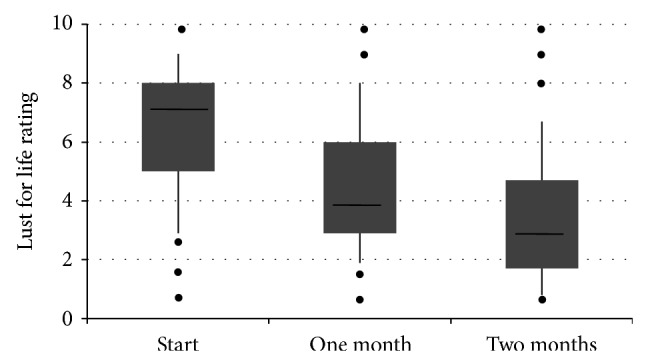
A box plot of the patients' rating of the impaired “lust for life” at start of treatment and after one month and 2 months treatment. The data shows the median (horizontal bar), 1st and 3rd quartiles (top and bottom of box, resp.), and the standard deviation (vertical bar). The dots represent single outlying values.

The significant improvements in pain and mobility of the patients during transdermal fentanyl were associated with similarly significant reductions in the patients' rating of their disturbed sleep and reduced QoL. After one month and 2 months of treatment, highly significant (*P* < 0.0001) decreases were recorded in the patients' perception of the levels of disturbed sleep (Table 4), reduced lust for life (Figure 3, Table 4), and reduced mood (Table 4). The mean relative declines in impairment of these parameters were similar to that of the functional parameters and also exceeded 30% at 2 months (Table 4).

### 3.6. Systemic Tolerability of the Transdermal Fentanyl (Fentavera) Matrix Patch

The physicians assessed the systemic tolerability of the transdermal fentanyl (Fentavera) matrix patch at one month and 2 months treatment and 86.38% and 91.03%, respectively, of cases exhibited “very good” or “good” systemic tolerance, while <3% exhibited “poor” or “very poor” tolerability.

### 3.7. Local (Skin) Tolerability of the Transdermal Fentanyl (Fentavera) Matrix Patch

The physicians assessed the local tolerability of the transdermal fentanyl (Fentavera) patch at one month and 2 months treatment. At both assessment intervals, more than 90% of cases exhibited “very good” or “good” local tolerance, while there were rare cases of “poor” or “very poor” tolerance.

### 3.8. Adhesiveness of the Transdermal Fentanyl (Fentavera) Matrix Patch

The physicians assessed the adhesiveness of the transdermal fentanyl (Fentavera) matrix patch at one month and 2 months treatment. At both assessment intervals, more than 87% of cases exhibited “very good” or “good” adhesiveness, while there were rare cases of “poor” or “very poor” adhesiveness.

### 3.9. Comedications

Table 5 lists the comedications taken by the patients at the start and during treatment. Analgesics were by far the most predominant comedication but the number of patients taking analgesics during the transdermal fentanyl (Fentavera) matrix patch treatment was drastically reduced compared to that at the start of treatment. The number of patients taking other comedications was also reduced during the treatment.

### 3.10. Adverse Events

Thirty adverse events were reported in 18 (4.2%) of patients (Table 6). The adverse events varied, with nausea (6 patients, 1.4%) and vomiting (5 patients, 1.2%) being the most frequent. Three patients (1.4%) had serious adverse events that were life-threatening and there was one fatality. The fatality was a female aged 88 years with noncancer pain (neuropathic and bone), receiving 50 mcg/h fentanyl (patch) and with prior fentanyl exposure; no further details were recorded. The 3 patients with life-threatening adverse events were all female and, in each case, the treatment-relatedness of the adverse event was not designated: one patient was aged 82 years with soft tissue pain after postoperative (hip operation) infection and had, as adverse event, “confused state” (previously experienced) of unrecorded duration or outcome (received 75 mcg, 100 mcg, and 125 mcg/h transdermal fentanyl); the second patient was aged 76 years with bone pain due to osteoarthritis and had, as adverse event, stomach pain that lasted 2 weeks (previously experienced) before recovery (received 25 mcg/h transdermal fentanyl); the third patient had asthma and multiple allergies, was aged 76 years with bone pain due to osteoarthritis, and had, as adverse event, stomach pain with an onset of eczema of the arms and abdomen of unrecorded duration before recovery (received 25 mcg, 50 mcg/h transdermal fentanyl).

## 4. Discussion

This large multicentre observational study provides a comprehensive real-life assessment of the effectiveness of the transdermal fentanyl (Fentavera) matrix patch in patients with chronic pain.

There are a few other noninterventional studies that have been published and these have shown the efficacy of transdermal fentanyl in cancer and noncancer pain [8, 34, 35]. Our findings are a significant addition to the data derived from previous clinical trials and noninterventional studies.

The patch tested in this study was a matrix patch designed to provide a reliably constant and fixed rate of delivery of fentanyl and contained an extract of* Aloe vera*, which is a common ingredient of skin cosmetics. The only selection criteria for patients entering the study were that they were adult and had chronic pain, and that their pain was responsive to opioids. Mostly, the patients had pain due to cancer or cancer treatment and had various sources of pain, the most frequent of which were bone (66.0%) and neural tissue (46.2%) (Table 2). A wide range of dosages of fentanyl were included in the study (Table 3), including a significant number of opioid-naïve patients who were started on the lowest dosage, 25 *μ*g/hour.

All patient-rated outcome parameters showed a similarly significant mean degree of improvement following transdermal fentanyl (Fentavera) treatment after one month (Table 4). For all parameters, the mean degree of improvement was greater than 30% at 2 months. There was a significant (*P* < 0.0001) decrease in pain intensity, as rated by the patients, and this was consistent with the physicians assessment that a large majority of patients (68%) exhibited a “good” response or better. Subanalyses (data not shown) revealed that, for all causes of pain, the mean pain reduction was at least 24% of baseline. The effective analgesia was also indicated by the drastically reduced number of patients taking analgesics during treatment with transdermal fentanyl (Fentavera) matrix patches compared to that at the start of the study (Table 5). The decreased pain intensity was paralleled by highly significant (*P* < 0.0001) decreases in impaired walking and impaired general activity.

Fentanyl is a well-established WHO-designated level 3 analgesic but its effectiveness in terms of improving QoL of the patient with chronic pain has not been extensively documented. Our findings indicate significant (*P* < 0.0001) improvements in both of the QoL outcomes that were assessed, “lust for life” and “mood.” Impaired sleep was also significantly (*P* < 0.0001) improved. Sleep is not often considered in health-related QoL surveys [36]. However, pain is associated with disturbed sleep [37] and sleep lowers pain thresholds [38] and sleep is now recognized as an important outcome measure in clinical trials of pain management [28].

For determining analgesic treatment effects, particularly in observational studies, 11-point numerical rating scales are often used to determine the degree of pain intensity [33, 39–41] and for rating quality of life [42]. A decrease on the rating scale of 2 points is generally considered to be clinically relevant [33]. It was found that a high proportion of our fentanyl-treated patients attained a degree of pain relief greater than 2 points (67% at one month and 75% at 2 months).

The physicians' assessments of the transdermal fentanyl (Fentavera) matrix patch indicated good or very good systemic (in close to 90% of patients) and local tolerance (in over 90% of patients) and a good or very good adhesiveness (in close to 90% of treatments), with rare cases of inadequate adhesiveness being reported. Adhesiveness is an important issue, not only for effective treatment and patient compliance but also in regard to preventing accidental opioid abuse. The overall safety of the transdermal fentanyl (Fentavera) patches was very good with few patients (4.2%) reporting any adverse events (Table 6).

The study has certain limitations. There was no statistical correction made for multiple outcomes. The study was purely noninterventional and so lacked any control arm and was not designed to analyze any placebo effect. Selection of patients and their treatment regime was solely the responsibility of the attending physician and there was no recording of possible opioid addiction and no systematic monitoring of compliance or possible treatment abuse.

## 5. Conclusion

The data presented here consists of a broad range of measures of effectiveness and clearly indicates that, in clinical practice, transdermal fentanyl is an effective and safe treatment for chronic pain management. In addition to positive effects on pain, mobility, sleep, and QoL, the proprietary transdermal fentanyl patch that was tested here was well tolerated and exhibited good or very good adhesiveness.

## Figures and Tables

**Table 1:** Conversion table for deriving the starting dosage of transdermal fentanyl from the prior daily oral dosage of opioid^*^ (expressed as morphine oral dosage equivalence).

Morphine oral dosage equivalence (mg per 24 hours)		Required transdermal fentanyl dosage (*μ*g per hour)
Patients needing opioid rotation [43]	Patients on stable, well-tolerated opioid treatment^**^	
<44	<60	12.5
45–134	60–89	25
135–224	90–149	50
225–314	150–209	75
315–404	210–269	100
405–494	270–329	125
495–584	330–389	150
585–674	390–449	175
675–764	450–509	200
765–854	510–569	225
855–944	570–629	250
945–1034	630–689	275
1035–1124	690–749	300

^*^For opioid-naïve patients, the starting dose of transdermal fentanyl was 25 *μ*g/hour; ^**^for patients who are not on opioid rotation but simply switching from oral to transdermal opioid treatment.

**(a):** 

*Patients *	
Sample size (number)	426
Female (number, %)	180, 42.25
Male (number, %)	238, 55.87
Unknown gender (number, %)	8, 1.88
Age (years) (*n* = 414)	
Mean ± sd	64.7 ± 14.4
Median	67.0
Range	24–96
Height (cms) (*n* = 415)	
Mean ± sd	169.6 ± 10.4
Median	170.0
Range	140–198
Weight (kg) (*n* = 416)	
Mean ± sd	75.5 ± 10.4
Median	73.0
Range	37–186

**(b):** 

Pain properties	Number	% of patients
Cause (*n* = 426)		
Cancer	122	28.6
Noncancer	127	29.8
Chemotherapy	28	6.6
Postoperative	50	11.7
Radiotherapy	25	5.9
Other	142	33.3
Missing data	23	5.4
Source^*^		
Bone	281	66.0
Neural tissue	197	46.2
Soft tissue	137	32.2
Visceral tissue	69	16.2

sd: standard deviation; ^*^several patients had pain from more than one source.

**Table 3:** The distribution (percentage of number of patients treated) of doses used at start and 1 and 2 months of treatment.

Dosage (mcg/hour)	% of study patients		
	Start	One month	Two months
25	46.48	23.24	25.19
50	31.69	28.64	26.18
75	12.21	16.20	15.21
100	6.81	8.92	12.72
No data	2.82	23.0	20.70

**Table 4:** Results of assessment of pain intensity and of various pain-related impairments: (i) mean VAS scores at baseline and at 1 and at 2 months; (ii) mean changes from baseline values of VAS scores at 1 and at 2 months; (iii) mean relative (%) changes from baseline values of VAS scores at 1 and at 2 months; (iv) 95% confidence intervals (CI) of differences from baseline values of mean VAS scores at 1 and at 2 months.

VAS score			VAS score change from baseline		VAS score relative (%) change over baseline		VAS score change from baseline	
(i)			(ii)		(iii)		(iv)	
Mean ± standard deviation			Mean ± standard deviation		Mean ± standard deviation		Mean, 95% CI	
Baseline	1 month	2 months	1 month	2 months	1 month	2 months	1 month	2 months
*n* = 425	*n* = 415	*n* = 398	*n* = 412	*n* = 392	*n* = 412	*n* = 392	*n* = 412	*n* = 392
Pain intensity								
6.68 ± 1.79	4.39 ± 1.99	3.59 ± 2.06	−2.27 ± 1.78	−3.06 ± 2.17	−33.4 ± 25.2	−44.9 ± 29.1	−2.27, −2.44; −2.09^*^	−3.06, −3.28; −2.85^*^

Impaired general activity								
6.57 ± 2.04	4.60 ± 2.18	3.85 ± 2.27	−1.94 ± 1.90	−2.70 ± 2.34	−27.5 ± 33.8	−38.3 ± 40.5	−1.94, −2.13; −1.76^*^	−2.70, −2.94; −2.47^*^

Impaired walking								
6.07 ± 2.42	4.30 ± 2.42	3.53 ± 2.40	−1.76 ± 2.02^b^	−2.54 ± 2.46	−27.1 ± 39.8^c^	−39.8 ± 42.6^f^	−1.76, −1.96; −1.57^b∗^	−2.54, −2.78; −2.29^*^

Impaired sleep								
5.79 ± 2.35	3.65 ± 2.17^a^	2.97 ± 2.03	−2.13 ± 2.15	−2.82 ± 2.56	−33.5 ± 39.1^d^	−43.8 ± 40.8^g^	−2.13, −2.34; −1.92^*^	−2.82, −3.08; −2.57^*^

Impaired lust for life								
6.49 ± 2.36	4.44 ± 2.29^a^	3.70 ± 2.19	−2.02 ± 2.20	−2.79 ± 2.61	−28.8 ± 32.7^e^	−38.9 ± 37.2^h^	−2.02, −2.23; −3.05^*^	−2.79, −3.04; −2.53^*^

Impaired mood								
6.38 ± 2.42	4.36 ± 2.30^a^	3.60 ± 2.26	−1.98 ± 2.25	−2.76 ± 2.69	−27.4 ± 45.0^e^	−38.7 ± 41.5^h^	−1.98, −2.20; −1.77^*^	−2.76, −3.03; −2.49^*^

^a^ *n* = 416; ^b^ *n* = 411; ^c^ *n* = 403; ^d^ *n* = 400; ^e^ *n* = 408; ^f^ *n* = 385; ^g^ *n* = 383; ^h^ *n* = 390; ^*^ *P* < 0.0001.

**Table 5:** List of types of comedications and number of patients using them at start and during one month and 2 months of treatment with transdermal fentanyl (Fentavera) patches.

Comedication type	Number of patients		
	Start	One month	2 months
Analgesics/anti-inflammatory	258	34	22
Psychoactive drugs	59	10	10
Antiepileptics	71	4	1
Neuropathic/neurotropic drugs	27	5	1
Corticosteroids (oral)	1	8	3
Unreadable	10	1	—
Undefined	4	3	—
Muscle relaxants	4	—	2
Gastrointestinal drugs	4	1	1
Migraine drugs	5	—	—
Laxatives	1	2	
Narcotics	2	—	1
Drugs for osteoporosis or calcium and bone metabolism	1	1	—
Sedatives	1	—	—
Antithrombotics	—	1	—
Diuretics	—	—	1

Total	465	65	40

**Table 6:** Summary of total adverse events during 2 months treatment with the transdermal fentanyl (Fentavera) patch.

	*n*
Preferred terms (MedDRA)	
Nausea	6
Vomiting	3
Vertigo	2
Restlessness	2
Abdominal pain upper	2
Fatigue	1
Sedation	1
Dysphoria	1
Retching	1
Feeling abnormal	1
Dyspnoea	1
Confusional state	1
Rash	1
Death	1
Dermatitis allergic	1
Pain	1
Diarrhoea	1
Swelling	1
Headache	1
Depressive symptom	1
System-Organ-Class (SOC) (MedDRA)	
Gastrointestinal disorders	13
General disorders and administration site conditions	5
Psychiatric disorders	5
Ear and labyrinth disorders	2
Nervous system disorders	2
Skin and subcutaneous tissue disorders	2
Respiratory, thoracic, and mediastinal disorders	1
Outcome	
Recovered	16
Unknown	1
Fatal	1
Life-threatening	3
Causality assessment	
Certain	4
Probable	9
Possible	2
Unlikely	3
